# Green Synthesis of Three-Dimensional Hybrid N-Doped ORR Electro-Catalysts Derived from Apricot Sap

**DOI:** 10.3390/ma11020205

**Published:** 2018-01-28

**Authors:** Ramesh Karunagaran, Campbell Coghlan, Cameron Shearer, Diana Tran, Karan Gulati, Tran Thanh Tung, Christian Doonan, Dusan Losic

**Affiliations:** 1 School of Chemical Engineering, University of Adelaide, Adelaide, SA 5005, Australia; ramesh.karunagaran@adelaide.edu.au (R.K.); diana.tran@adelaide.edu.au (D.T.); k.gulati@griffith.edu.au (K.G.); tran.tung@adelaide.edu.au (T.T.T.); 2 School of Chemistry, University of Adelaide, Adelaide, SA 5005, Australia; cam.coghlan@adelaide.edu.au (C.C.); christian.doonan@adelaide.edu.au (C.D.); 3 School of Chemical and Physical Sciences, Flinders University, Adelaide, SA 5042, Australia; cameron.shearer@flinders.edu.au

**Keywords:** oxygen reduction reaction (ORR), catalysis, carbon nanotubes, carbo microsphere, N–doped carbon

## Abstract

Rapid depletion of fossil fuel and increased energy demand has initiated a need for an alternative energy source to cater for the growing energy demand. Fuel cells are an enabling technology for the conversion of sustainable energy carriers (e.g., renewable hydrogen or bio-gas) into electrical power and heat. However, the hazardous raw materials and complicated experimental procedures used to produce electro-catalysts for the oxygen reduction reaction (ORR) in fuel cells has been a concern for the effective implementation of these catalysts. Therefore, environmentally friendly and low-cost oxygen reduction electro-catalysts synthesised from natural products are considered as an attractive alternative to currently used synthetic materials involving hazardous chemicals and waste. Herein, we describe a unique integrated oxygen reduction three-dimensional composite catalyst containing both nitrogen-doped carbon fibers (N-CF) and carbon microspheres (N-CMS) synthesised from apricot sap from an apricot tree. The synthesis was carried out via three-step process, including apricot sap resin preparation, hydrothermal treatment, and pyrolysis with a nitrogen precursor. The nitrogen-doped electro-catalysts synthesised were characterised by SEM, TEM, XRD, Raman, and BET techniques followed by electro-chemical testing for ORR catalysis activity. The obtained catalyst material shows high catalytic activity for ORR in the basic medium by facilitating the reaction via a four-electron transfer mechanism.

## 1. Introduction

The continued rise in global energy demand and the depletion of the world’s non-renewable resources has initiated a global push towards renewable energy sources. Among the most promising methods for producing renewable energy are fuel cells, which have emerged as a promising avenue of research due to their ability to generate high power density [1]. Fuel cells are devices that electrochemically combine gaseous fuel (e.g., hydrogen) and an oxidant gas (e.g., oxygen) to produce electricity and heat by an oxygen reduction reaction (ORR) [1]. The efficiency of fuel cells and their practical applicability is dependent on the ORR catalyst present in the cell [2]. However, slow kinetics have hindered fuel cells from being utilised outside of a laboratory environment [3]. Currently, platinum (Pt) catalysts have outperformed all other catalysts in areas such as activity, stability, and selectivity [4] and have dominated the fuel cell industry as the preferred ORR catalysts [5,6,7]. However, these catalysts have been overlooked for industrial scale-up due to their high cost and low availability [8]. To overcome this problem, non-precious transition metals (Fe, Co, Ni) in addition to hetero atoms have been trialed to enhance the ORR activity [9,10,11,12,13]. The transition metals have the ability to significantly increase ORR catalytic activity by facilitating the incorporation of hetero atoms, such as nitrogen, in the carbon matrix during pyrolysis [14,15,16,17]. The incorporation of electro-negative nitrogen into a graphitic carbon framework has shown to induce high positive charge density on adjacent carbon atoms [18]. The electron donor properties of nitrogen-doped adjacent carbon atoms trigger a favourable diatomic O_2_ adsorption and ultimately weaken the O_2_ bond strength to facilitate ORR activity [15,18,19,20,21].

The high electrical conductivity of the mesoporous carbon materials [22] and their metal oxide hybrids has been utilised in applications such as lithium-ion batteries [23,24], super capacitors [25,26], and catalysts [27,28,29] in recent years. Various chemical approaches have been developed to synthesise nitrogen-doped carbon materials for ORR catalysis utilising materials such as graphene [30] and carbon nanotubes (CNTs) [31]. Carbon materials doped with nitrogen precursors, such as melamine (C_3_H_6_N_6_) [32,33], ethylene diamine (C_2_H_4_(NH_2_)_2_) [34], o-phenylenediamine (C_6_H_8_N_2_) [11], and ammonia (NH_3_) [35], have shown high ORR activity. Mesoporous N-doped carbon spheres synthesised using multiple different methods, such as the one-pot soft template method [22], spray pyrolysis [36,37], and self-polymerisation [38], each show outstanding catalytic potential for ORR catalysis. However, high cost, hazardous chemical usage and waste has limited their translation into scale-up industrial applications [22,36,37,38].

To address this problem, green chemistry approaches using low-cost natural materials to synthesise mesoporous carbon (e.g., plant *Typha orientalis* [39], catkin [40], lignin [41], and soya chunks [42]) have been successfully demonstrated as efficient ORR catalysts. Apricot trees (*Prunus armeniaca* L.) are widely cultivated in areas where a scarcity of water remains the main obstacle for cultivation [43]. In many regions, apricot trees suffer from gummosis, a bark disease [44] resulting in the formation of sap which oozes out from the wounds caused from factors including weather, infection, insects, or mechanical damage. The sap commonly appears as an amber-coloured material, which contains various sugar components, such as xylose, arabinose, rhamnose, glucose, mannose, and galactose [45]. Analysis performed by Lluveras et al. [45] revealed that apricot sap consists of polysaccharides, primarily arabinose and galactose. Polysaccharides, such as galactose, glucose, sucrose, and starch, have been shown to undergo dehydration and subsequent aromatisation when hydrothermally treated at 160–200 °C, resulting in their conversion to char material with nano- or micrometer-size smooth carbon spheres [27,46].

Carbon microspheres (CMS) have recently attracted attention due to their unique applications, high density, and high strength in carbon product fabrication [47]. Carbon-spheres synthesised using polysaccharides have successfully been implemented in the application of catalysis for synthetic fuel [27], Li-ion batteries [48], and electrochemical capacitors [49]. Previously, hybrid CMS containing transition metals have been synthesised using polysaccharides [27,46]. The ability of the iron oxides to bind with the oxygen functional groups in the sugar molecules through coulombic and/or electrostatic interactions has resulted in the formation of iron oxide encapsulated carbon spheres [27]. Similar hybrid materials can be synthesised using the polysaccharides present in apricot sap, which have not been used for any catalytic application in the past.

**Scheme 1:**
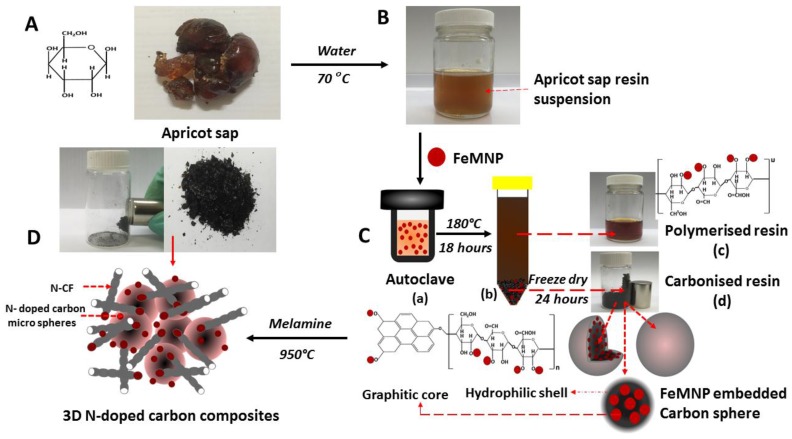
Schematic procedure of three-dimensional (3D)-integrated N-doped carbon microspheres (CMS) and N-doped carbon fibers N-CFs catalysts from apricot sap. (**A**) Apricot sap collected from the apricot tree; (**B**) apricot sap dissolved in water (apricot resin suspension); (**C**) apricot resin suspension containing FeMNP, hydrothermally treated to produce magnetic insoluble char material (carbonised resin) with FeMNP embedded CMS structures (HT-APG-Fe); and (**D**) HT-APG-Fe pyrolised with melamine to form N-doped integrated structures containing N-CFs and N-CMS (N-APG-Fe). FeMNP: iron oxide magnetic nanoparticle.

This work explores the use of apricot sap containing sugar molecules as a natural source and method for the generation of a new type of three-dimensional (3D) hybrid N-doped ORR electro-catalysts composed of microspherical and nanotubular structures. These catalysts were synthesised through a three-step process as shown in Scheme 1. Firstly, an apricot sap resin suspension containing polysaccharides of arabinose and galactose was prepared. Secondly, the apricot resin solution was hydrothermally treated with iron oxide nanoparticle or cobalt precursors to obtain a char material with carbon microspheres embedded with magnetic nanoparticles. Finally, the char material was pyrolysed (950 °C) with a nitrogen precursor of melamine to dope the graphitic carbons with nitrogen. The pyrolysed composite material forms an integrated composite material with both carbon fibers (CFs) and CMS. A similar integrated structure was reported in our previous paper, where we hypothesised that the decomposition of melamine during pyrolysis causes disruption to the iron oxide magnetic nanoparticle clusters’ (FeMNPC) surface that is embedded in the carbon sphere to diffuse FeMNPC particles out of the sphere to catalyse the formation of N-doped carbon fibers (N-CFs) [50]. This hybrid carbon catalyst contains N-CFs and N-doped carbon microspheres (N-CMS) with magnetic nanoparticles, forming a unique 3D intergrated morphology.

The structural and chemical composition of the prepared N-doped 3D integrated catalyst with FeMNPs (N-APG-Fe) and cobalt nanoparticle clusters (CoMNPs) (N-APG-Co) were characterized with SEM, TEM, XRD, Raman, and BET followed by testing their electrochemical catalytic properties and ORR activity. The conversion process of the naturally occurring waste and apricot sap material into an effective electro-catalyst for ORR reaction is also described.

## 2. Results and Discussion

### 2.1. Formation of Integrated Morphology of N-CFs and N-CMS

The steps involved in the synthesis of N-APG-Fe are shown in Scheme 1. In the first step, apricot sap (Scheme 1A) was dissolved in water (70 °C) to make a translucent light-orange colour resin suspension (Scheme 1B). In the second step, the resin suspension was hydrothermally treated in the presence of FeMNPs. During this process, the oxygen functional groups (i.e., OH and C=O) bind to the iron oxide particles through Coulombic interactions to form a hybrid material [27]. During the hydrothermal process (Scheme 1(Ca)), sugar molecules polymerise (Scheme 1(Cb)) to form intermolecular crosslinks between linear or branched oligosaccharides due to dehydration [46]. As a result of dehydration and polymerisation, oxygen functional groups associated with the sugar molecules were reduced along with their negative charges to make the polymerised material more water-insoluble. Subsequently, the insoluble material (char) settles as FeMNPs-embedded spheres with a hydrophobic core and hydrophilic shell [46,51,52] (Scheme 1(Cc)). In the final process, the hydrothermally obtained char is pyrolysed in the presence of melamine at 950 °C to introduce N atoms into the carbon framework. The pyrolysed composite material forms an integrated composite material with both N-CF and N-microspheres (Scheme 1D). During pyrolysis, the decomposition of melamine caused disruption to the spheres’ surface and caused the FeMNPCs embedded within the sphere to diffuse out [50], which catalysed the formation of N-CF [53]. The synthesised hybrid material, which consists of both N-CF and N-CMS, formed a unique 3D integrated morphology.

### 2.2. Structual and Chemical Characterisation of Prepared 3D N-Doped Carbon Composites

**Figure 1:**
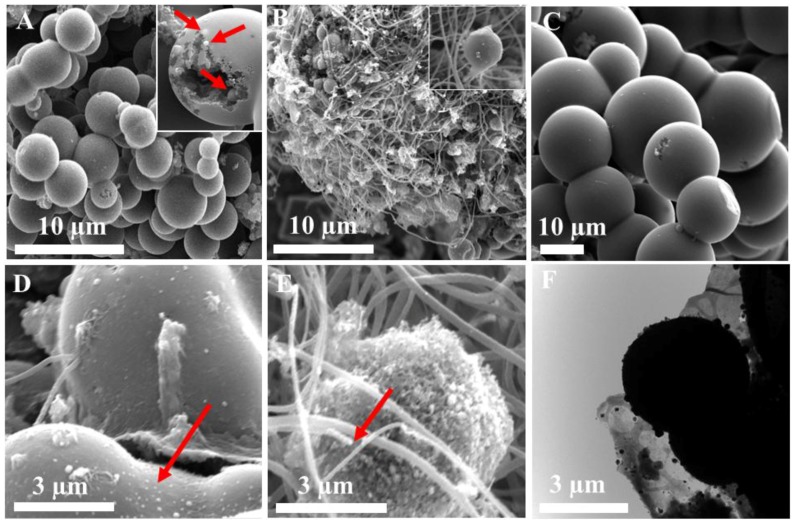
SEM images of (**A**) carbon microspheres formed from char material of hydrothermally treated apricot resin (HT-APG-Fe), (**B**) integrated structure composed of CMS and CFs of HT-APG-Fe pyrolysed with melamine at 950 °C (N-APG-Fe) (inset shows the presence of micro spheres and CFs), (**C**) carbon micro spheres of HT-APG-Fe pyrolysed without melamine at 950 °C (APG-Fe), (**D**) formation of CFs from FeMNPC from the sphere interior of N-APG-Fe (red arrow shows FeMNPC diffused out of the sphere after pyrolysis with melamine), (**E**) formation of CF from FeMNPC diffused out of the sphere in N-APG-Fe (red arrow shows the CF forming from the tip of FeMNPC), and (**F**) TEM image of FeMNPC diffused out of the sphere in N-APG-Fe.

The morphology of the hydrothermally induced char material (HT-APG-Fe) was determined using SEM (Figure 1), which shows the formation of carbon microspheres (1–6 μm). The image of a broken sphere (Figure 1A, inset) shows FeMNPCs embedded within the microspheres. EDX analysis conducted on the particles (Figure S1 in supporting information (SI)) indicated that an average of 57% (wt %) of the material consisted of Fe, confirming the presence of FeMNPCs in the carbon sphere. The magnetic property of the material was confirmed by applying an external magnet to the sample.

In order to make HT-APG-Fe catalytically active, it was pyrolysed with a N precursor (melamine) (N-APG-Fe) at 950 °C to introduce N atoms into the carbon framework and improve catalytic properties (Figure 1B). The nitrogen doping eliminates the electro-neutrality of the carbon framework and generates favourable charged sites for oxygen adsorption [19,54]. Similarly, to compare the catalytic activity of the N-doped and non N-doped catalysts, HT-APG-Fe was pyrolysed without melamine (APG-Fe) at 950 °C and the SEM image is presented in Figure 1C. The SEM revealed that APG-Fe formed interconnected smooth microspheres in the range of 1–6 μm. Interestingly, in contrast to the smooth CMS formed in APG-Fe (Figure 1C), the catalysts pyrolysed with melamine (N-APG-Fe) (Figure 1B) formed an integrated composite material with both CF and CMSs. EDX analysis was performed to determine the N-doping on CMS and CF and revealed an average of 2.55 and 2.04 (At %) of N presented in the CMS and the CF, respectively. During pyrolysis, the decomposition of melamine causes disruption to the FeMNPC’s surface that is embedded in the carbon sphere for the FeMNPC particles to diffuse out of the sphere and form N-CFs (Figure 1D,F).

To demonstrate if this synthetic procedure is generic for the formation of 3D integrated N-CMS and N-CF structures, we repeated the procedure using cobalt precursors, which is commonly used as an alternate transition metal to fabricate ORR catalysts. The SEM images of the hydrothermally produced structures (Figure S2A), pyrolysed with and without melamine (Figure S2B,C), respectively, revealed that integrated structures with carbon spheres and CFs, similar to N-APG-Fe, were produced when the hydrothermally reduced char materials containing cobalt oxide nanoparticles were pyrolyzed with melamine. The hydrothermally produced char material without any nanoparticles (HT-APG, Figure S3A), when pyrolysed with melamine (Figure S3B), did not produce any integrated products of CMS and CFs. As the integrated structures were only seen on the catalysts with transition metals (Figure 1B and Figure S2B in SI), we deduce that both a transition metal oxide and a nitrogen precursor are needed for the synthesis of the integrated structure comprised of both N-CMS and N-CFs. Previously, we reported a 3D integrated structure of N-CMS and N-CFs using sugar galactose (N-GAL-Fe) [50]. A similar morphology was observed in N-APG-Fe, which demonstrates that the presence of galactose in the apricot sap also contributes significantly to the formation of the integrated structure.

**Figure 2:**
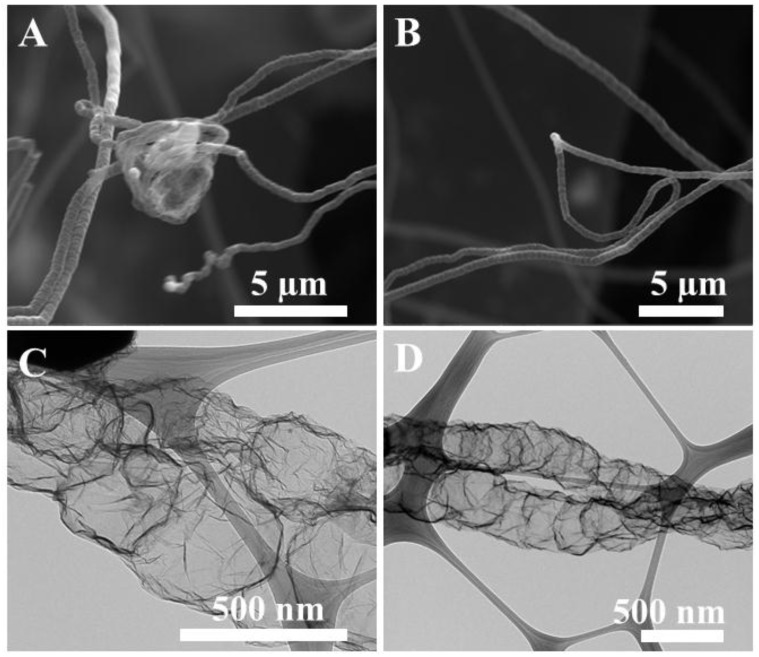
SEM images of N-CF obtained from (**A**) N-APG-Fe and (**B**) N-APG-Co. TEM image of (**C**) N-APG-Fe and (**D**) N-APG-Co.

The morphologies of N-CF in N-APG-Fe and N-APG-Co (Figure 2A,B) were further investigated with TEM. The images clearly illustrate that the N-CFs originate from the tip of the MNPC. An EDX analysis was conducted on the particles at the tip of the CF (Figure 2A,B) and revealed 59.20% and 18.50% (wt.%) of Fe and Co, respectively, suggesting that the CFs are formed by a metal-induced mechanism [53]. The TEM images of CF from N-APG-Fe (Figure 2C) and N-APG-Co (Figure 2D) show that the CFs possess an irregular corrugated morphology with a width of approximately 150–500 nm, similar to those reported by M. Terrones et al. [55]. To investigate the presence of N-doping on these CFs (which facilitate ORR) [31], an EDX elemental analysis was performed on CFs grown from Fe (Fe-CF) and Co (Co-CF). The N-content was found to be 2.04 and 6.32 At. % for Fe-CF and Co-CF, respectively, compared to 0% in the non-doped sample, confirming nitrogen doping on CF. This reveals that C and N precursors from pyrolysed melamine had diffused into the metal clusters to form the CF [53,56].

**Figure 3:**
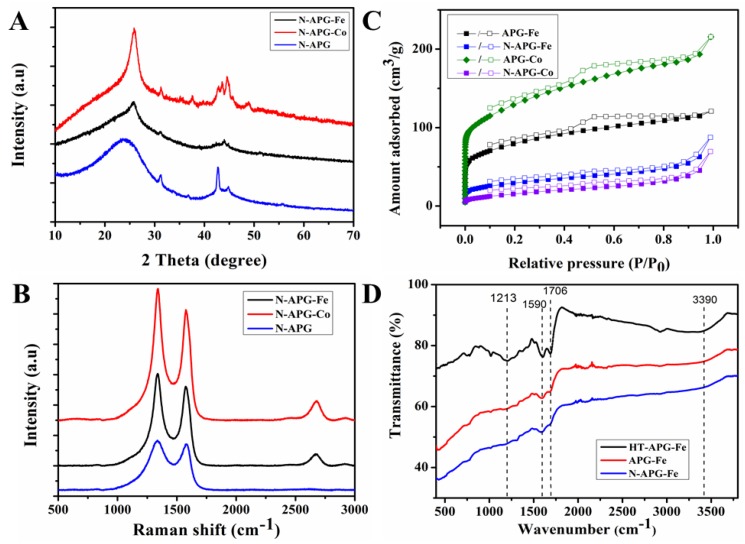
(**A**) XRD spectrum of N-APG, N-APG-Fe, and N-APG-Co, (**B**) Raman spectrum of N-APG, N-APG-Fe, and N-APG-Co, (**C**) N_2_ adsorption/desorption isotherm of APG-Fe, N-APG-Fe, N-APG-Co, and N-APG-Co, (**D**) FTIR spectrum of HT-APG-Fe, APG-Fe, and N-APG-Fe.

The XRD analysis conducted on N-APG, APGFe-N, and APGCo-N is shown in Figure 3A. The diffraction peaks seen at 25.78°, 42.66°, and 44.83° for APGFe-N and APGCo-N correspond to diffraction facets (002), (110), and (101), respectively, assigned to the presence of graphitic carbon [57,58,59]. Similarly, N-APG showed peaks at 23.96°, 42.53°, and 44.67° for diffraction facets (002), (110), and (101), respectively. The positive shift of the (002) peak of N-APG from 23.96° to 25.78° in N-APG-Fe and N-APG-Co can be assigned to the formation of the graphitic crystalline structure induced by the reduction of the oxygen functional group containing sugar molecules with a metal MNPC [60].

The Raman spectrum performed on N-APG, N-APG-Fe, and N-APG-Co is shown in Figure 3B. N-APG-Fe and N-APG-Co show three characteristic peaks at 1342, 1581, and 2684 cm^−1^ for the D, G, and 2D bands, respectively, while N-APG showed only the D and the G band at 1338 and 1583 cm^−1^, respectively. The additional 2D band indicates the presence of crystalline graphitic carbon material formed during the annealing process. This was facilitated by the reduction of oxygen groups in the sugar molecules by the addition of FeMNPC [61,62]. The I_D_/I_G_ of N-APG (1.07), N-APG-Fe (1.16), and N-APG-Co (1.13) shows the disruption of sp^2^ bonds and the formation of sp^3^ defect sites [63,64], which are associated with the N-doping on the carbon framework. The higher I_D_/I_G_ for N-APG-Fe and N-APG-Co compared to N-APG revealed that the transition metal particles have facilitated the incorporation of N atoms to the carbon framework to distort the graphitic framework [15,65]. This shows that the addition of transition metals formed greater positive sites on the adjacent carbon atoms to adsorb oxygen, thus enhancing the ORR activity.

The N_2_ sorption isotherms of non-doped APG-Fe and APG-Co differ from the doped N-APG-Fe and N-APG-Co (Figure 3C). The characteristic type IV isotherm and H4 hysteresis loop for APG-Fe and APG-Co shows the presence of mesoporous slip-like pores [62,66,67] with mean pore size distributions of 4.64, 5.62, 7.68, and 13.84 Å (Figure S4). The surface area of the prepared catalysts was measured using Brunauer-Emmett-Teller (BET) and is shown in Table 1. The surface area measured for the doped catalysts was much lower than that of the non-doped catalysts. We hypothesise that the mesopores on the surface of the carbon microspheres on the non-doped catalysts contributed to the higher surface area. The significant reduction in the surface area of the doped sample can be assigned to the disruption of these mesopores or blocked pores due to the decomposition of melamine during pyrolysis.

The presence of any carbonyl groups was analysed by FTIR, which can form condensation products with melamine. The FTIR spectra of hydrothermally treated (HT-APG-Fe), pyrolysed without melamine (APG-Fe), and N-doped (N-APG-Fe) materials are presented in Figure 3D. HT-APG-Fe showed characteristic peaks at 1213, 1590, 1706, and 3390 cm^−1^ [68,69,70], which can be attributed to C-O and C-H stretching, the stretching vibration of C=O, carbonyl vibrations, and the stretching vibration of O-H, respectively. The presence of oxygen functional groups suggests that the carbon spheres were formed with a hydrophilic shell containing oxygen groups as suggested by Sun et al. [46] and Mer et al. [54]. When the peaks corresponding to the carbonyl groups of doped N-APG-Fe and non-doped APG-Fe were compared, a reduction of the intensity of the N-APG-Fe was observed. Since carbonyl groups can interact with the amine group of melamine [71], we hypothesised that the melamine was attached to the carbon spheres before undergoing complete decomposition. It is likely that these condensation products caused surface disruption of the microspheres and studies need to be undertaken to confirm this hypothesis.

XPS measurements were performed to determine the nitrogen species present in the N-APG-Fe catalyst. The high-resolution XPS C 1s spectrum (Figure S5) showed a variety of carbon bonds, including C-C (285.04 eV), C-N (286.03 eV), and O-C=O (290.03 eV) [72,73,74]. The high-resolution N 1s XPS spectra displayed in Figure S5B showed three distinct peaks, including pyridine-N (398.38 eV), graphitic-N (401.35 eV), and nitrogen oxide-N (403.12 eV) [75,76]. The high percentage (45.57 At. %) of pyridinic N (in the N1s analysis) along with 63.03 At. % of C-N (in the C1s analysis) in N-APG-Fe clearly demonstrates efficient N-doping on the carbon framework to facilitate O_2_ adsorption. Similar peaks for C 1s and N 1s were observed for N-APG-Co (Figure S6). The N-doping on the graphitic carbon framework altered the electro-neutrality of the nano-carbon material. The pyridinic nitrogen with its strong electronic affinity induced high positive charge density on the adjacent carbon atoms. Thus, the electron donor properties of the N-doped adjacent carbon atoms are favourable for weakening the strength of the O-O bond to facilitate ORR activity [2,19,77]. However, the XPS analysis did not show any presence of Fe or Co. This showed that Fe-N-C sites were not formed and only N-C carbon has been formed.

### 2.3. Electrochemical Characterisation of Catalytic Performance

The electrochemical activity of N-doped (N-APG, N-APG-Fe, and N-APG-Co) catalysts were examined by cyclic voltammetry (CV) (Figure S7). The voltammograms between 0 and 1.2 V show well-defined cathodic peaks centered at 0.55, 0.67, and 0.74 V, respectively. The voltammograms of N-APG-Fe and N-APG-Co showed a higher positive overpotential shift, 120 and 190 mV, respectively, compared N-APG. These results indicate that the hybrid structures of N-CF and N-CMS formed by the introduction of Fe and Co have aided to increase ORR activity and O_2_ uptake. Since the XPS did not detect any Fe-N-C active sites, these enhancements of ORR activity can be attributed to N-C catalytic sites merely in both the N-CF and N-CMS in the hybrid structure in N-APG-Fe and N-APG-Co.

**Figure 4:**
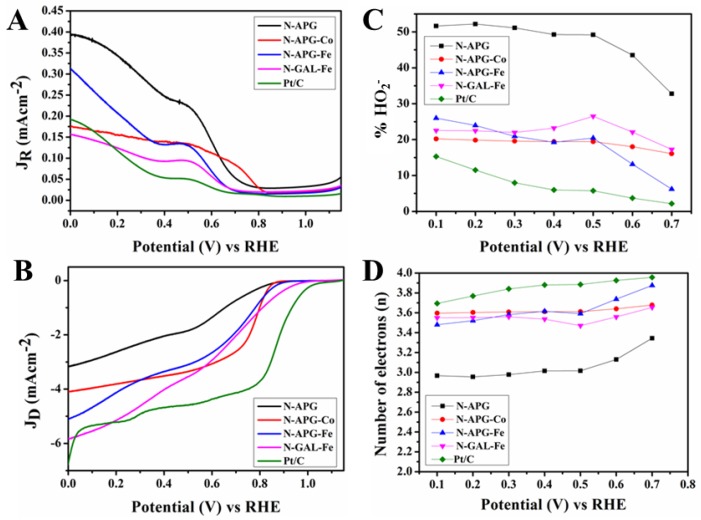
Rotating ring disc voltammograms of (**A**) ring current, (**B**) disc current of N-APG, N-APG-Co, N-APG-Fe, N-GAL-Fe, and Pt/C electrodes in oxygen-saturated 0.10 M KOH at 2000 rpm at a scan rate of 10 mV/s. (**C**) Percentage HO_2_- and (**D**) number of electrons of N-APG, N-APG-Fe, N-APG-Co, and Pt/C electrodes at various potential calculated according to RRDE data. RHE: reversible hydrogen electrode

To understand the reaction kinetics of N-APG, N-APGFe and N-APGCo, Rotating Ring Disc Electrode(RRDE) was employed to quantify the overall electron transfer number (n) and percentage of hydrogen peroxide (% HO_2_^−^). To explore the dependence on galactose sugar in the electron transfer kinetics, N-GAL-Fe was contrasted against these catalysts in the potential range between 0.10–1.15 V and the ring (Figure 4A) and disc (Figure 4B) currents. The onset potential measured for these catalysts (Table 2) showed a positive shift for N-APG-Fe (0.88 V) and N-APG-Co (0.86 V) compared to N-APG (0.84 V), revealing that the hybrid structures have initiated the ORR faster. However, the half-wave potential (E_1/2_) of all of these catalysts shifted negatively compared to the standard Pt/C. The negative shift in the E_1/2_ is due to the absence of any M-N-C catalytic active sites present in the catalysts. Liu et al. [78] described that M-N-C active sites perform the ORR reaction with a more positive E_1/2_ compared to N-C active sites in the catalysts.

The number of electrons transferred using N-APG, N-APG-Co, N-APG-Fe, and N-GAL-Fe catalyst electrodes within the potential region 0.10–0.70 V is shown in Table 2. The electron transfer number towards four of these catalysts reveals that the ORR reaction is carried out predominantly via a four-electron transfer mechanism. The catalytically analysed values and comparison chart of n and % HO_2_^−^ at 0.40 V (Figure S8) shows that both of the N-doped apricot and galactose catalysts follow a similar trend, showing the significance of galactose in the electron transfer mechanism. Unlike the doped catalysts, the non-doped catalysts did not perform effectively (Figure S9). The electro-chemical properties summarised in Table S1 in the SI of these catalysts showed a negative onset potential and lower electron transfer numbers than the doped catalysts. This shows that the doping of nitrogen has created more catalytically active sites for ORR. The stability of the N-APG-Co and N-APG-Fe was determined by cycling the catalysts between 0.00 V and 1.15 V at 100 mVS^−1^ in an oxygen-saturated 0.1 M KOH solution (Figure S10 in the SI). The results show that after 6000 cycles the onset overpotential had increased by 30 mV and 40 mV for N-APG-Co and N-APG-Fe, respectively, indicating only a slight deterioration of the catalysts.

**Figure 5:**
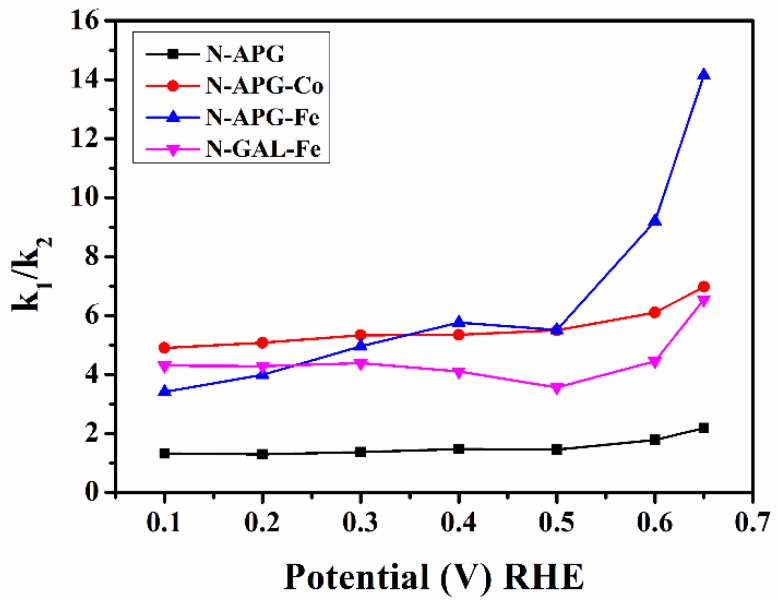
The ratio of rate constant k1/k2 for, N-APG, N-APG-Fe, N-APG-CO and N-GAL-Fe in the potential range of 0.10–0.65V.

The kinetics of electron transfer using the details obtained from RRDE and the scheme suggested by Damjanovic et al. [79] are described in the SI. The rate constants were calculated based on these equations for the N-APG, N-APG-Co, N-APG-Fe, and N-GAL-Fe in the potential region of 0.10–0.65 V (Figure S11 in SI). The calculated rate constants showed that N-APG-Co, N-APG-Fe, and N-GAL-Fe were predominantly driven by a four-electron k_1_ kinetics, while in N-APG, the ORR was carried out via both the k_1_ and k_2_ pathways. The calculated value of the ratio of k_1_/k_2_ presented in Table S2 in the SI and Figure 5 showed k_1_/k_2_ >1 for all catalysts. The higher values of k_1_/k_2_ for N-APG-Co, N-APG-Fe, and N-GAL-Fe compared to N-APG showed a dominant four-electron k_1_ electron transfer pathway for these catalysts that demonstrates that the presence of the hybrid structure of N-CF and N-CMS significantly contributes to the generation of the active sites for oxygen adsorption.

In order to compare the efficiency of the N-doped apricot catalysts and galactose catalysts, the materials were contrasted against similar catalysts, and the comparison is presented in Table S3 in the SI. The comparison revealed that these catalysts had similar levels of activity compared with other synthetic material presented in the literature. While the performance of these materials is lower than the highest-performing Pt catalysts, the advantages of this approach are the scalable, stable, low-cost, and natural non-hazardous starting materials and the ease of their synthesis. However, the use of apricot sap in industrial large-scale production may be limited by its low yield. Our previous paper demonstrated the synthesis of a similar hydride structure comprising N-CF and N-CMS to fabricate C-N electrodes for ORR using galactose as the source. This approach has the potential to be implemented to synthesise C-N electrodes with similar integrated hybrid structures using natural and synthetic feedstocks containing polysaccharides. Furthermore, the production of efficient ORR catalysts at a lower cost to current catalysts, using natural resources and environment-friendly processes, may provide a step forward for natural products.

## 3. Materials and Methods

### 3.1. Materials

Apricot sap from an Apricot Moorpark tree (*Prunus armeniaca*) (South Australia) was collected from a local garden. Iron (II) chloride tetra hydrate (FeCl_2_·4H_2_O) (Sigma Aldrich, St Louis, MO, USA), iron (III) chloride hexa hydrate (FeCl_3_·6H_2_O) (Chem Supply, Gillman, Australia), hydrochloric acid (HCl) (Chem Supply, Gillman, Australia), ammonia (Chem Supply, Gillman, Australia), cobalt (II) acetate (Sigma Aldrich, St Louis, MO, USA), melamine (Sigma Aldrich, St Louis, MO, USA), and platinum standard catalyst (20 w% Vulcan XC-72) were used as purchased.

### 3.2. Methods

#### 3.2.1. Synthesis of Carbonaceous Spheres from Apricot Sap (HT-APG)

The apricot sap (cca 100 g) was cut by a knife from a tree. The sap was washed with fresh water and dried in open air for 12 h. The sap (25 g) was dissolved in water (100 mL) and heated to 70 °C with manual stirring. The resin suspension was sealed and left for 24 h in an open environment. The obtained transparent light orange suspension was then filtered to obtain a contaminant-free resin suspension. The resin suspension (50 mL) was transferred in to an autoclave and heated at 180 °C for 18 h. The char was centrifuged and washed with distilled water (6 × 35 mL). The washed char was then freeze dried for 24 h. The final product weighed 1.62 g.

#### 3.2.2. Synthesis of Cobalt Embedded Carbonaceous Spheres (HT-APG-Co)

Cobalt (II) acetate (150 mg) was dissolved with 50 mL of filtered resin suspension and stirred for 30 min. The product was then transferred to an autoclave and heated for 18 h at 180 °C. The product was cooled to room temperature and transferred in to a centrifuge tube and centrifuged with repeated washing with distilled water for six times (6 × 35 mL) and four times with 0.5 M H_2_SO_4_. The washed char was then freeze-dried for 24 h.

#### 3.2.3. Synthesis of Maghemite Nanoparticles

Maghemite nanoparticles were synthesised according to the previously established method [80]. Briefly, FeCl_2_·4H_2_O (39.76 g) and FeCl_3_·6H_2_O (16.29 g) were dissolved in 1 M HCl (100 mL). The solution was stirred for 2 h and the pH adjusted to 9.8 using 2 M ammonia solution. Finally, the product was centrifuged and washed three times with distilled water (35 mL) and once with ethanol (35 mL) and dried for 6 h at 60–70 °C.

#### 3.2.4. Synthesis of Fe-Embedded Carbonaceous Spheres (HT-APG-Fe)

Maghemite nanoparticles (200 mg) were suspended in the filtered resin suspension (50 mL) and stirred for 30 min, transferred to an autoclave, and heated for 18 h at 180 °C. The product was collected and centrifuged by washing with distilled water (6 × 35 mL) and four times with 0.5 M H_2_SO_4_. The product was then freeze-dried for 24 h and denoted as HT-APG-Fe.

#### 3.2.5. Synthesis of Fe-Embedded Carbonaceous Spheres with Galactose (HT-GAL-Fe)

Maghemite nanoparticles (200 mg) were added into a suspension of 0.02 mole galactose in 40 mL water and mixed with stirring for 30 min. The mixture was transferred in to a Teflon autoclave and heated up to 180 °C for 18 h. Then, the product was collected, centrifuged, and repeatedly washed, six times with deionised water and four times with 0.5 M H_2_SO_4_. The product was collected and freeze-dried for 24 h (referred to as HT-GAL-Fe).

#### 3.2.6. Pyrolysis of Carbonaceous Spheres with N-Precursor (N-Doped Carbon Spheres)

Each of the hydrothermally treated samples HT-APG, HT-APG-Fe, HT-APG-Co, and HT-GAL-Fe were ground together with melamine (1:10 *w*/*w*) using a mortar and a pestle. The mixture was placed in a tubular furnace and pyrolysed at 950 °C for 3 h under Ar at the rate of 10 °C/min. The N-doped products are referred to as N-APG, N-APG-Fe, N-APG-Co, and N-GAL-Fe, respectively.

#### 3.2.7. Pyrolysis of Carbonaceous Spheres without N-Precursor

Hydrothermally synthesised HT-APG, HT-APG-Fe, HT-APG-Co, and HT-GAL-Fe were individually pyrolysed at 950 °C for 3 h under Ar at the rate of 10 °C/min in the tubular furnace. The pyrolysed products are referred to as APG, APG-Fe, APG-Co, and GAL-Fe, respectively.

#### 3.2.8. Preparation of Catalytic Inks

Catalytic ink was prepared by ultra-sonication of each catalyst (2 mg) and suspended in Nafion suspension (1 mL of 1%). The prepared ink (10 µL) was carefully deposited on a glassy carbon rotating disc electrode (3 mm) and a rotating ring disc electrode (4 mm). The sample was then allowed to dry in air for 12 h.

### 3.3. Characterization

Scanning electron microscopy (SEM) images and energy-dispersive X-ray spectroscopy (EDX) were obtained using a Quanta 450 (FEI, Hillsboro, OR, USA) at an accelerating voltage of 10 kV. For EDX, three readings were obtained and the average was recorded. Transition electron microscopy (TEM) investigation was carried out using a Tecnai G2 Spirit (FEI, Hillsboro, OR, USA) operated at 120 kV. X-ray diffraction (XRD) was performed at 40 kV and 15 mA in the range of 2θ = 10–70° at a speed of 10°/min using a Miniflex 600 (Rigaku, Akishima, Tokyo, Japan). Gas adsorption isotherms were conducted using a Micromeritics 3-Flex or ASAP2020 analyser (Micro metrics Instruments Corporation, Norcross, GA, USA). The Brunauer–Emment–Teller (BET) surface area and pore size distribution were calculated using software on the Micromeritics 3-Flex or ASAP 2020 analyser (Beckman Coulter, Indianapolis, IN, USA). Fourier transform infrared (FTIR) spectroscopy was conducted using Spectrum 100 (Perkin Elmer, Waltham, MA, USA). Raman analysis was conducted using a LabRAM Evolution (Horiba Yvon, Kyoto, Japan) using a 532 nm wavelength. XPS was conducted on a custom-built SPECS instrument (Berlin, Germany). All XPS (X-ray photo electron spectroscopy) measurements were performed on sample prepared by drop-casting onto Si using a non-monochromatic Mg source operating at 120 kV and 200 W. High resolution XPS spectra were collected using a pass energy of 10 eV with an energy step of 0.1 eV.

#### Electrochemical Characterization

The ORR reactions were conducted utilising a Rotating Ring Disc Electrode (RRDE) apparatus connected to a bi potentiostat (CH 1760 C, CH Instruments Inc., Austin, TX, USA) in a standard three-electrode cell with an oxygen-saturated KOH (0.1 mol/L) solution. The glassy carbon electrode, platinum, and reversible hydrogen electrode (RHE) were used as the working, counter, and reference electrodes, respectively. The scan rate of the reaction was 0.01 Vs^−1^ in the range of 0 and 1.1 V. The cycle was repeated until stable voltammograms were obtained before the RRDE readings were derived at different speeds from 400 to 2400 rpm.

The reaction kinetics of the catalysts were examined by employing RRDE to quantify the overall electron transfer number (*n*) and percentage of hydrogen peroxide (% HO_2_^−^) at rotation speeds from 400 to 2400 rpm in an oxygen-saturated 0.1 M KOH solution. To elucidate the overall number of electrons (*n*) and % HO_2_^−^ produced in the ring against the applied potential, Equations (1) and (2) were employed [10,81].

(1) $$n=\frac{4I_{D}}{I_{D}\;+\;\frac{I_{R}}{N}}$$

(2) $$\%H_{2}O_{2}=100\;\frac{2I_{R}}{I_{D}N+\;I_{R}}$$

where *I_D_* and *I_R_* are the disc and ring currents, respectively, and *N* is the collection efficiency.

## 4. Conclusions

The phenomenon of converting a naturally occurring apricot sap from an apricot tree into a 3D hybrid ORR electro-catalyst composed of N-CF and N-CMS is reported and verified by SEM and TEM. The MNPs initially embedded within the CMS diffused out of the CMS to catalyse for the formation of corrugated hollow N-CF due to the surface destruction caused by the decomposition of melamine during pyrolysis. The 3D integrated N-CMSs and N-CF ORR electro-catalysts prepared using FeNP (N-APG-Fe) or CoNP (N-APG-Co) showed a predominant four-electron transfer pathway for the ORR within the potential region of 0.10–0.70 V. The spherical morphology obtained from non-hazardous apricot sap can be employed in a wide range of areas, such as catalysis applications, absorption studies, and drug delivery.

## Figures and Tables

**Table 1:** Surface area of doped and non-doped apricot catalysts with Fe and Co.

Catalyst	Surface Area (m^2^/g)
APG-Fe	235.38
N-APG-Fe	73.15
APG-Co	375.62
N-APG-Co	39.86

**Table 2:** Surface area of doped and non-doped apricot catalysts with Fe and Co.

Product	Current density (mA/cm^2^) at 0 V (RHE)	Onset Potential (RHE) (V)	Number of Electrons (n) (0.10–0.70 V) (RHE)	% HO_2_^−^ (0.10–0.70 V) (RHE)
N-APG	3.05	0.84	2.96–3.34	51.64–32.77
N-APG-Co	4.03	0.86	3.59–3.67	20.16–16.08
N-APG-Fe	4.91	0.88	3.48–3.87	25.99–6.19
N-GAL-Fe	5.81	0.96	3.54–3.65	22.54–17.25
Pt/C	6.70	1.04	3.69–3.95	15.20–2.15

## Supplementary Materials

The following are available online at http://www.mdpi.com/1996-1944/11/2/205/s1, Figure S1: EDX analysis of FeMNPC, Figure S2: SEM images of (A) hydrothermally treated apricot sap resin and cobalt acetate (HT-APG-Co), (B) pyrolysed HT-APG-Co at 950 °C with the presence of nitrogen precursor melamine (N-APG-Co), and (C) pyrolysed HT-APG-Co at 950 °C without melamine (APG-Co), Figure S3: SEM images of (A) hydrothermally treated apricot sap resin (HT-APG), (B) pyrolysed HT-APG at 950 °C with the presence of nitrogen precursor melamine (N-APG), and (C) pyrolysed HT-APG at 950 °C without melamine (APG), Figure S4: Pore size distribution of (A) APG-Fe and (B) APG-Co, Figure S5: XPS core level spectra of N-APG-Fe for (A) C1s and (B) N1s, Figure S6: XPS core level spectra of N-APG-Co for (A) C1s and (B) N1s, Figure S7: Cyclic Voltammetry of (A) N-APG, (B) N-APG-Fe, and (C) N-APG-Co at a scan rate of 10 mVS-1 in oxygen-saturated 0.1M KOH solution, Figure S8: (A) Comparison of number of electrons and (B) % HO_2_^−^ of N-APG, N-APG-Co, N-APG-Fe, N-GAL-Fe, and Pt/C catalysts electrodes at 0.4 V applied potential in oxygen-saturated 0.10 M KOH electrolyte at 2000 rpm at a scan rate of 10 mV/s, Figure S9: Rotating ring disc voltammograms of (A) ring current and (B) disc current of catalysts electrodes APG, APG-Co, APG-Fe, GAL-Fe, and Pt/C, pyrolysed without the presence of melamine in oxygen saturated 0.1 M KOH at 2000 rpm at a scan rate of 10 mV/s. (C) Percentage peroxide, and (D) number of electrons of APG, APG-Fe, APG-Co, and Pt/C electrodes at various potential calculated according to RRDE data, Figure S10: RDE polarisation curves of (A) N-APG-Co and (B) N-APG-Fe with a scan rate of 100 mVS^−1^ before and after 6000 potential cycles in an oxygen saturated KOH solution, Figure S11: Rate constants of (A) N-APG, (B) N-APG-Co, (C) N-APG-Fe, and (D) N-GAL-Fe, Table S1: Electro chemical properties of non-doped apricot sap and galactose catalysts, Table S2: Comparison of performance of N-APG-Fe, N-APG-Co, and N-GAL-Fe with other similar carbon-based catalysts.
