# Supplementary material for: Green Synthesis of Three-Dimensional Hybrid N-Doped ORR Electro-Catalysts Derived from Apricot Sap
Source: Materials (Basel). 2018 Jan 28;11(2):205. doi: 10.3390/ma11020205 (PMC5848902; doi:10.3390/ma11020205)
Supplement: Supplementary file 1 [file materials-11-00205-s001.pdf]

Supplementary Information

Green Synthesis of Three-Dimensional Hybrid N-Doped ORR Electro-Catalysts Derived from Apricot Sap

Ramesh Karunagaran [1], Campbell Coghlan [2], Cameron Shearer [3], Diana Tran [1], Karan Gulati [1], Tran Thanh Tung [1], Christian Doonan [2] and Dusan Losic [1,*]

[1] School of Chemical Engineering, University of Adelaide, SA 5005, Australia; ramesh.karunagaran@adelaide.edu.au (R.K.); diana.tran@adelaide.edu.au (D.T.); k.gulati@griffith.edu.au (K.G.); tran.tung@adelaide.edu.au (T.T.T.)

[2] School of Chemistry, University of Adelaide, SA 5005, Australia; cam.coghlan@adelaide.edu.au (C.C.); christian.doonan@adelaide.edu.au (C.D.)

[3] School of Chemical and Physical Sciences, Flinders University, SA 5042, Australia; cameron.shearer@flinders.edu.au

* Correspondence: dusan.losic@adelaide.edu.au; Tel.: +61-8-8013-4648

Received: 10 January 2018; Accepted: 26 January 2018; Published: 28 January 2018

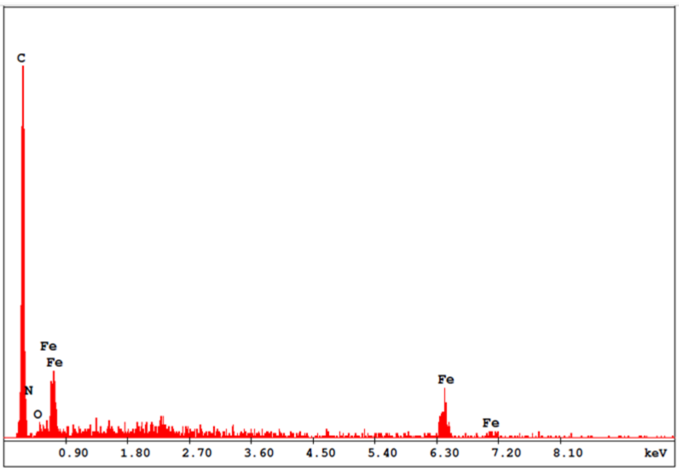

Figure S1. EDX analysis of FeMNPC embedded in the CMS.

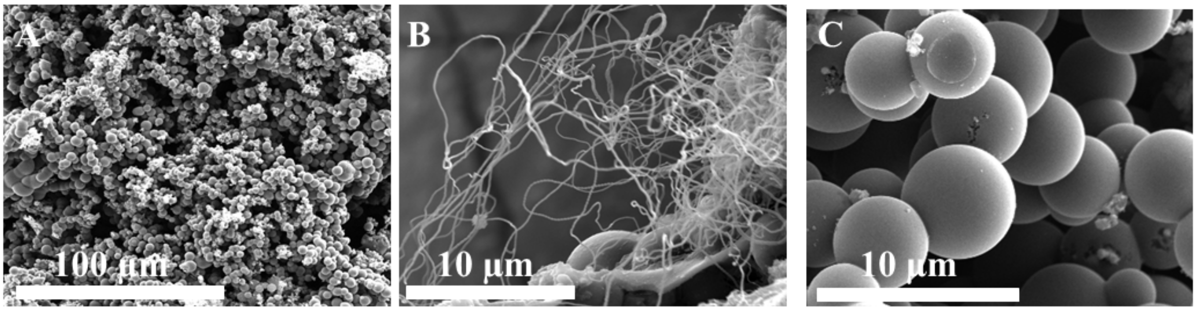

Figure S2. SEM images of (A) hydrothermally treated apricot sap resin and cobalt acetate (HT-APG-Co), (B) pyrolysed HT-APG-Co at 950°C with the presence of nitrogen precursor melamine (N-APG-Co), and (C) pyrolysed HT-APG-Co at 950°C without melamine (APG-Co)

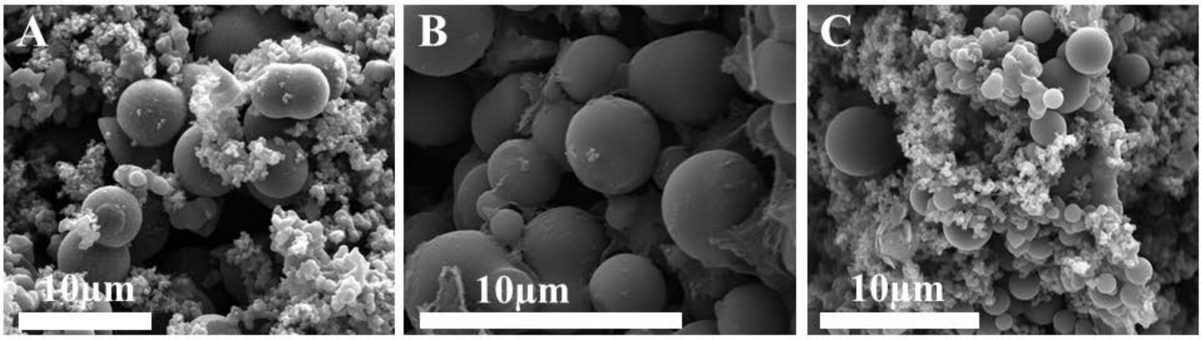

Figure S3. SEM images of (A) hydrothermally treated apricot sap resin (HT-APG), (B) pyrolysed HT-APG at 950°C with the presence of nitrogen precursor melamine (N-APG), and (C) pyrolysed HT-APG at 950°C without melamine (APG)

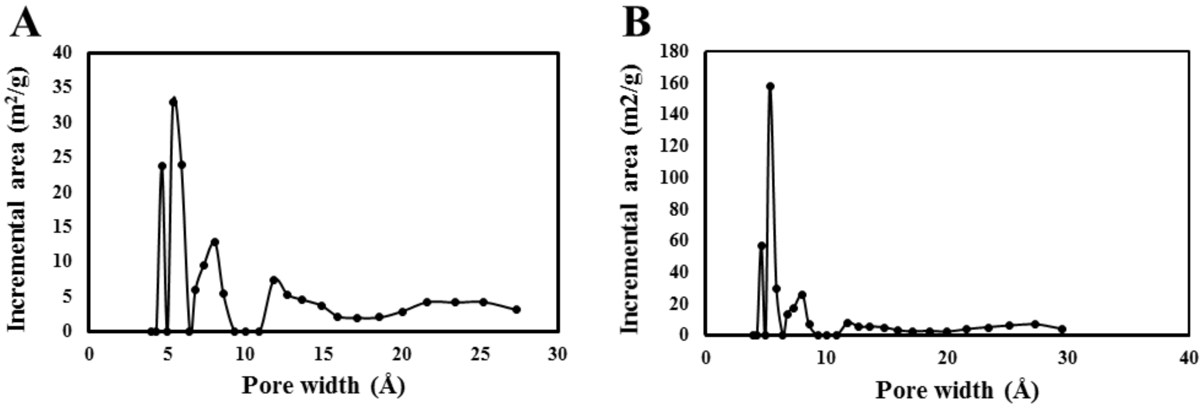

Figure S4. Pore size distribution of (A) APG-Fe and (B) APG-Co.

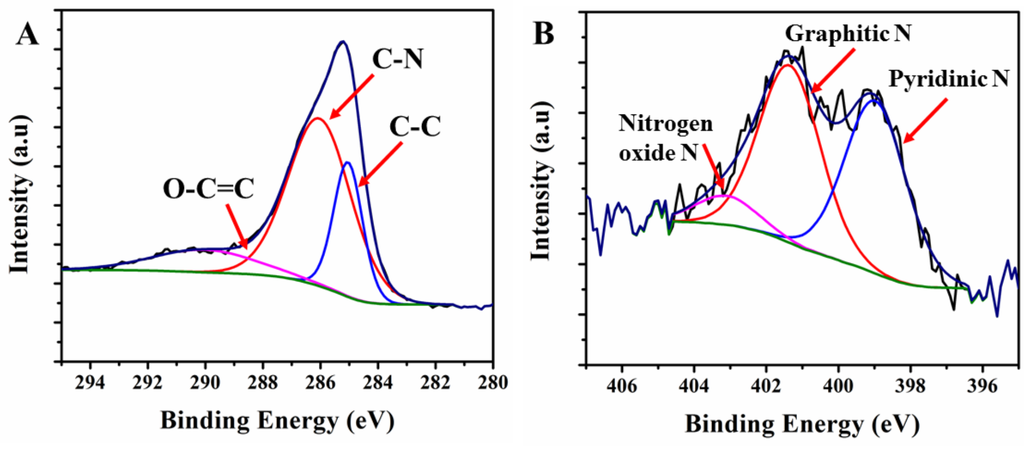

Figure S5. XPS core level spectra of N-APG-Fe for (A) C1s and (B) N1s.

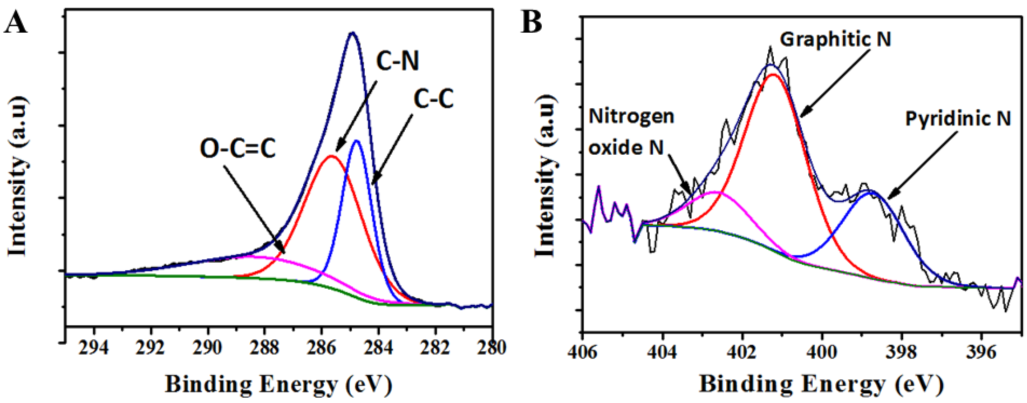

Figure S6. XPS core level spectra of N-APG-Co for (A) C1s and (B) N1s.

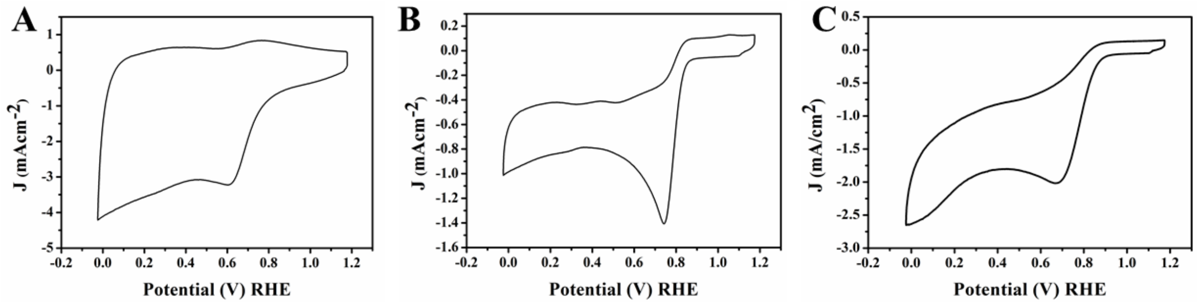

Figure S7. Cyclic Voltammetry of (A) N-APG, (B) N-APG-Fe and (C) N-APG-Co at a scan rate of 100 mVS-1 in oxygen saturated 0.1M KOH solution.

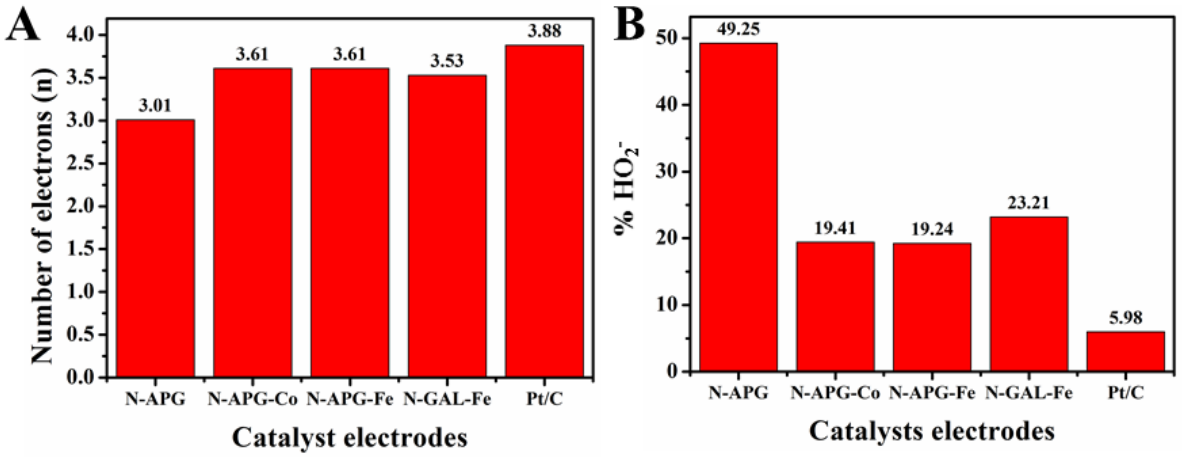

Figure S8. (A) Comparison of number of electrons and (B) % HO_2^- of N-APG, N-APG-Co, N-APG-Fe, N-GAL-Fe and Pt/C catalysts electrodes at 0.4V applied potential in oxygen saturated 0.10 M KOH electrolyte at 2000 rpm at a scan rate of 10 mV/s.

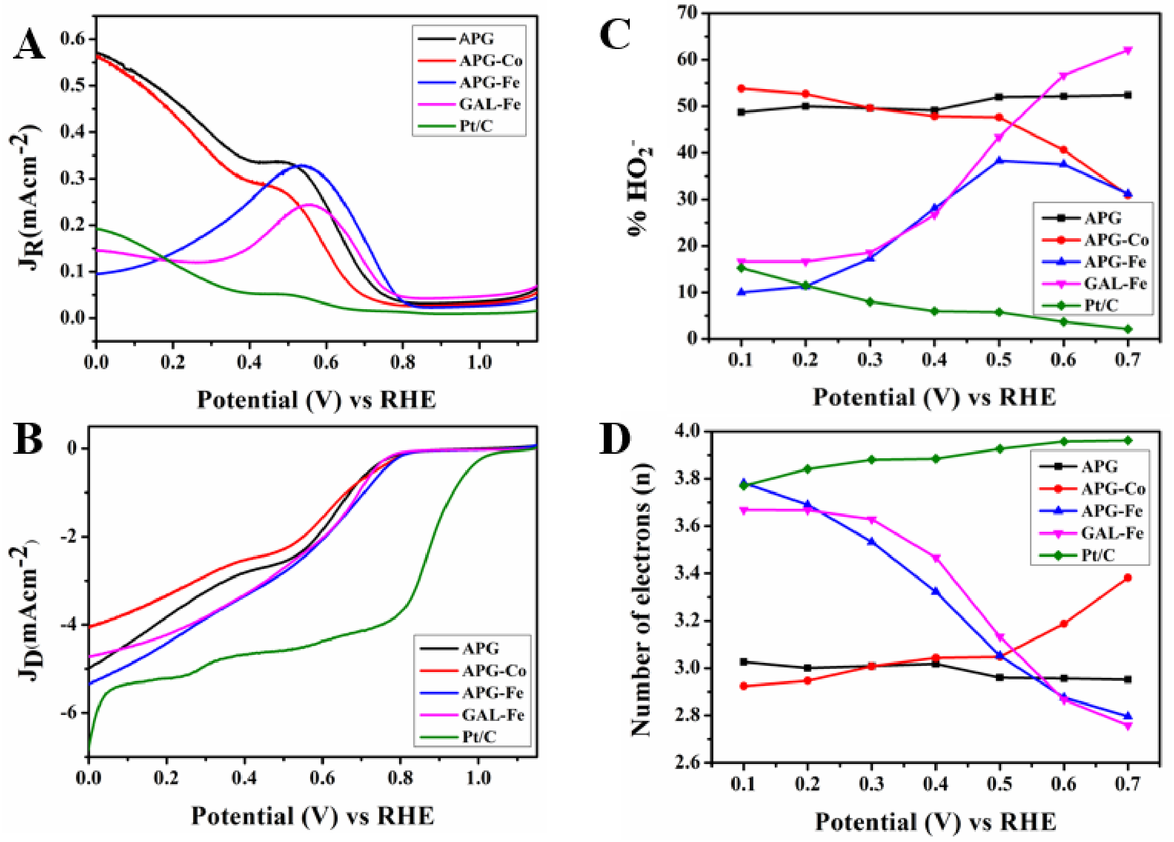

Figure S9. Rotating ring disc voltammograms of (A) ring current and (B) disc current of catalysts electrodes APG, APG-Co, APG-Fe, GAL-Fe and Pt/C, pyrolysed without the presence of melamine in oxygen saturated 0.1M KOH at 2000 rpm at a scan rate of 10mV/s. (C) Percentage peroxide, and (D) number of electrons of APG, APG-Fe, APG-Co and Pt/C electrodes at various potential calculated according to RRDE data.

Table S1. Electro chemical properties of non-doped apricot sap and galactose catalysts.

**Product**	**Current density (mA/cm^2) at 0V**	**Onset potential (V) (RHE)**	**Number of electrons (n) (0.1-0.7 V)**	**% HO_2^- (0.1-0.7V)**
APG	4.98	0.78	3.02-2.95	48.70-52.41
APG-Fe	5.33	0.80	3.78-2.79	10.90-60.21
APG-Co	4.05	0.80	2.92-3.38	53.81-30.91
GAL-Fe	4.72	0.82	3.66-2.75	16.60-62.10

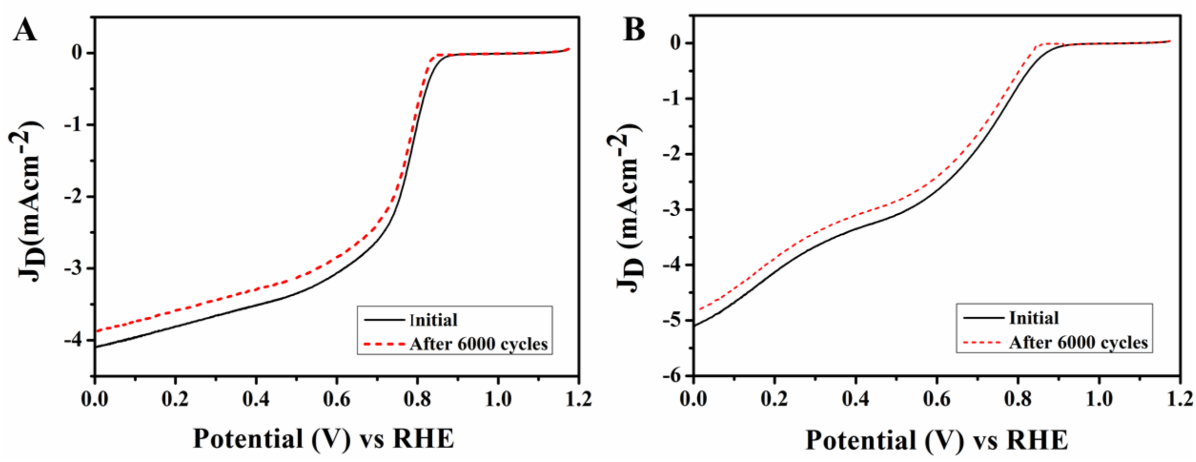

Figure S10. RDE polarisation curves of (A) N-APG-Co and (B) N-APG-Fe with a scan rate of 100 mVS^{-1} before and after 6000 potential cycles in an oxygen saturated KOH solution.

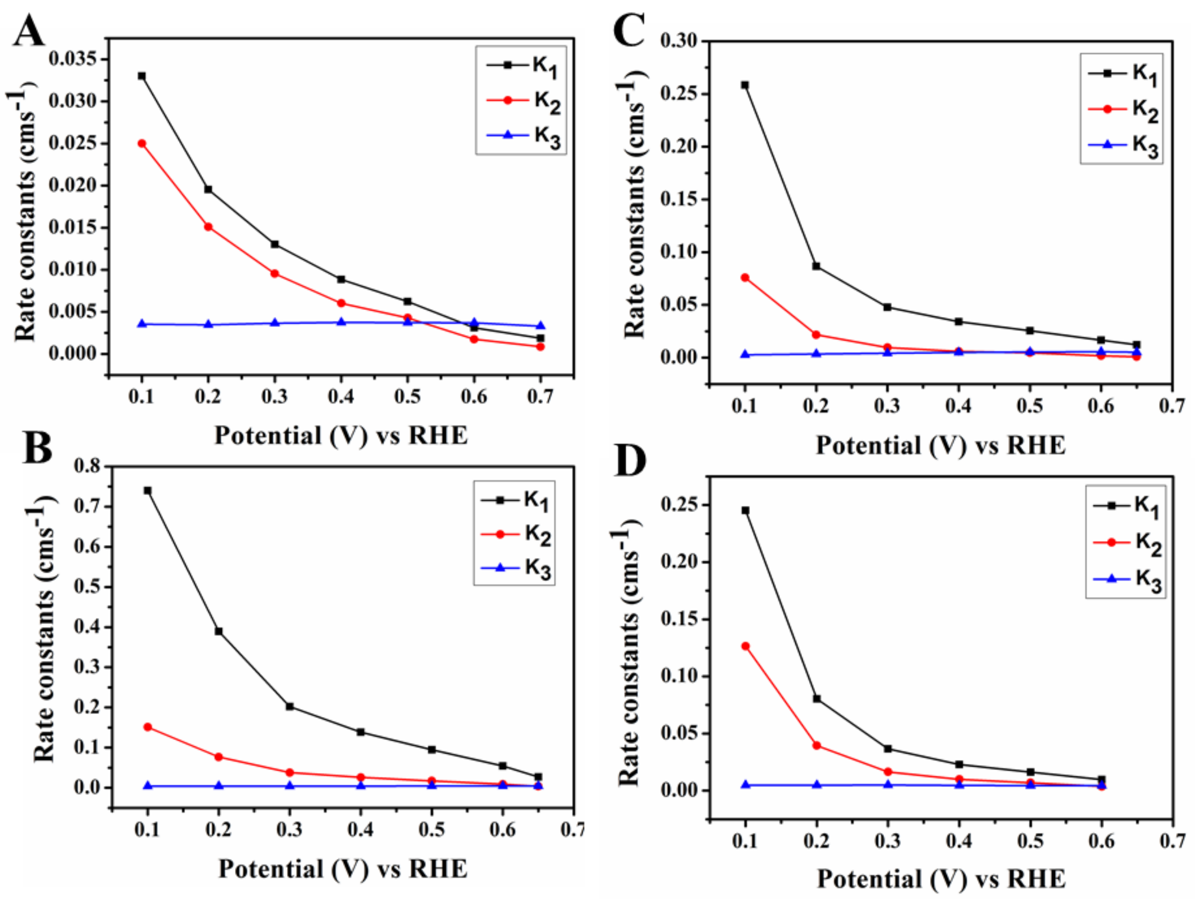

Figure S11. Rate constants of (A) N-APG, (B) N-APG-Co, (C) N-APG-Fe, and (D) N-GAL-Fe.

Table S2. comparison of k1/k2 of N-doped apricot and galactose catalysts

	k_1/k_2	
	**Potential 0.1V (RHE)**	**Potential 0.65V (RHE)**
N-APG	1.32	2.18
N-APG-Co	4.90	6.97
N-APG-Fe	3.40	14.14
N-GAL-Fe	4.30	6.40

Table S3. comparison of performance of N-APG-Fe, N-APG-Co and N-GAL-Fe with other similar carbon-based catalysts.

**Material**	**Onset potential (V)**	**Number of electrons (n) / Potential (V) (RHE)**	**Reference**
Soya -derived heteroatom doped carbon	0.96	3.70 / 0.625 V	[1]
N-doped mesoporous carbon spheres	0.86	3.40 / 0.575 V	[2]
N-doped hollow carbon spheres	0.80	3.82 / 0.575 V	[3]
Co-N-C hybrid using soya milk	0.80	3.70 / 0.675 V	[4]
3D-Integrated N-doped carbon sphere with N-CNT (N-GAL-Fe)	0.96	3.55 / 0.600 V	[8]
3D-Integrated N-doped carbon sphere with N-CF (N-APG-Co)	0.86	3.63 / 0.600 V	**This study**
3D-Integrated N-doped carbon sphere with N-CF (N-APG-Fe)	0.88	3.73 / 0.600 V	**This study**

Electron transfer kinetics

The electron transfer kinetic of the ORR was identified using RRDE voltametry (Scheme S1) [5, 6]. According to Damjanovic *et al.* [5] the electron transfer mechanism follows a direct four-electron pathway via k_1 kinetics (Scheme S1), in which oxygen is diectly reduced to hydroxide anion (OH^-) or could be driven through a two-electron pathway via k_2 kinetics producing peroxide intermediates (HO_2^-), followed by reduction to hydroxide anion (OH^-) through another two electron pathway through K_3 kinetics.

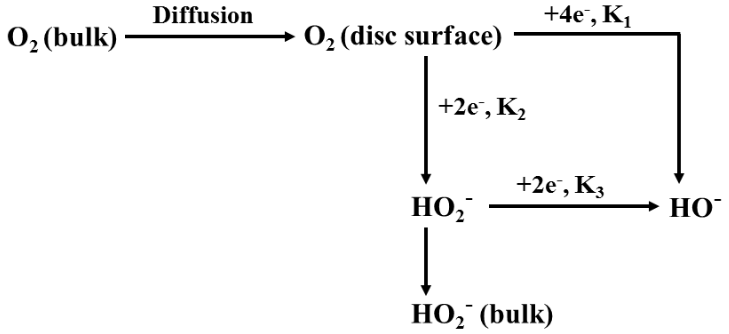

Scheme S1. Proposed model for electrochemical reduction of oxygen proposed by Damjanovic et al. and Hsueh et al.

Hsueh *et al.*[6] suggested a series of equations (3, 4 and 5) to calculate the rate constants K_1, K_2 *and* K_3., where Id, Ir, IdL and ω are the disc current, ring current, limiting disc current and the rotation speed, respectively.

$$k_1 = S_1 Z_1 \frac{I_1 N - 1}{I_1 N + 1} \quad (3)$$

$$k_2 = \frac{2\, S_2 Z_1}{I_1 N + 1} \quad (4)$$

$$k_3 = \frac{N S_1 Z_2}{I_1 N + 1} \quad (5)$$

Where S_1 and I_1 are the slope and intercept correspond to the I_d / I_r vs $\omega^{-1/2}$ plots and S_2 and is the slope of I_{dL} / I_{dL} – I_d vs $\omega^{-1/2}$ plot. $Z_1 = 0.62 D_{O_2}{}^{2/3}\, V^{-1/6}$, $Z_2 = 0.62 D_{H_2O_2}{}^{2/3}\, V^{-1/6}$, $D_{H_2O_2}$ is 6.8×10^{-6} $cm^2\ s^{-1}$ and N is the collection efficiency [7].

References

[1] Rana, M,; Arora, G.; Gautam, U. K. N-and S-doped high surface area carbon derived from soya chunks as scalable and efficient electrocatalysts for oxygen reduction. *Sci. Technol. Adv. Mater*. **2015**, *16*, 014803.

[2] Tang, J.; Liu, J.; Li, C.; Li, Y.; Tade, M. O.; Dai, S.; Yamauchi,Y. Synthesis of Nitrogen-Doped Mesoporous Carbon Spheres with Extra-Large Pores through Assembly of Diblock Copolymer Micelles. *Ang. Chem. Int. Ed*. **2015**, *54*, 588–593.

[3] Li, Y.; Li, T.; Yao,M.; Liu, S. Metal-free nitrogen-doped hollow carbon spheres synthesized by thermal treatment of poly (o-phenylenediamine) for oxygen reduction reaction in direct methanol fuel cell applications. *J. Mater. Chem.* **2012**, *22*, 10911–10917.

[4] Zhai, Y.; Zhu, C.; Wang, E.; Dong, S. Energetic carbon-based hybrids: green and facile synthesis from soy milk and extraordinary electrocatalytic activity towards ORR. *Nanoscale* **2014**, *6*, 2964–2970.

[5] Damjanovic, A.; Genshaw, M. A.; Bockris, J. O. Distinction between intermediates produced in main and side electrodic reactions. *J. Chem. Phys*. **1966**, *45*, 4057–4059.

[6] Hsueh, K.L.; Chin, D.L.; Srinivasan, S. Electrode kinetics of oxygen reduction: A theoretical and experimental analysis of the rotating ring-disc electrode method. *J. Electroanal. Chem. Interfacial Electrochem.* **1983**, *153*, 79–95.

[7] Muthukrishnan, A.; Nabae, Y.; Chang, C. W.; Okajima, T.; Ohsaka, T. A high-performance Fe and nitrogen doped catalyst derived from diazoniapentaphene salt and phenolic resin mixture for oxygen reduction reaction. *Catal. Sci. Technol*. **2015**, *5*, 1764–1774.
